# Infant mortality and social causality: Lessons from the history of Britain’s public health movement, c. 1834–1914

**DOI:** 10.1111/1468-4446.13121

**Published:** 2024-06-15

**Authors:** Elias Nosrati, Michael P. Kelly, Simon Szreter

**Affiliations:** ^1^ University of Oslo Oslo Norway; ^2^ University of Oxford Oxford UK; ^3^ Fafo Institute for Labour and Social Research Oslo Norway; ^4^ University of Cambridge Cambridge UK

**Keywords:** Arthur Newsholme, industrial capitalism, infant mortality, public health movement, social causality

## Abstract

What are the historical conditions under which a sociologically informed understanding of health inequality can emerge in the public sphere? We seek to address this question through the lens of a strategically chosen historical puzzle—the stubborn persistence of and salient variation in high infant mortality rates across British industrial towns at the dawn of the previous century—as analysed by Arthur Newsholme, the Medical Officer of the Local Government Board. In doing so, we retrace the historical processes through which the evolving public health movement gradually helped crystallise a scientific understanding of the social causes of excess mortality. We map the dominant ideology of the public sphere at the time, chart the shifting roles of the state, and retrace the historical origins and emergence of ‘public health’ as a distinctive category of state policy and public discourse. We situate the public health movement in this historical configuration and identify the cracks in the existing ideological and administrative edifice through which this movement was able to articulate a novel approach to population health—one that spotlights the political economy of social inequality. We relate this historical sequence to the rise of industrial capitalism, the social fractures that it spawned, and the organised counter‐movements that it necessitated.

## 1 INTRODUCTION

What are the historical conditions under which a sociologically informed understanding of health inequality can emerge in the public sphere, to the point where it palpably influences government policy? We seek to address this question through the lens of a strategically chosen historical puzzle, namely the stubborn persistence of, and salient variation in, high infant mortality rates across British industrial towns at the dawn of the previous century. We approach this puzzle from the unique perspective of a central public health figure—Arthur Newsholme, Medical Officer of the Local Government Board, 1908–19, and author of several major reports on infant and child health in Edwardian Britain—who sought to understand the social causes of excess mortality.

We map the dominant ideology of the public sphere at the time, chart the shifting roles of the state, and retrace the historical origins and emergence of ‘public health’ as a distinctive category of state policy and public discourse. We situate Newsholme and others, in this historical configuration and identify the cracks in the then existing ideological and administrative edifice through which they were able to articulate a novel approach to population health—one that spotlights the political economy of social inequality. We relate this historical sequence to the rise of industrial capitalism, the social fractures that it spawned, and the organised counter‐movements that it necessitated. Britain's industrial towns and their analysis by influential public officials, such as Newsholme, constitute a ‘strategic research site’ (Merton, 1987) insofar as they uniquely exemplify, in a manner ripe with lessons for the present, the causal confluence of multilevel forces that shape population health: individual lifeworlds, modes of social and economic organisation, attendant inequalities, and collectively articulated responses thereto.

## 2 BACKDROP: ‘NATIONAL EFFICIENCY’ AND POPULATION HEALTH

To comprehend the scale of the revolution in thinking towards a sociological understanding that took place in public policy in the ‘Edwardian’ era, c.1901–14, it is necessary to appreciate that throughout the long reign of Queen Victoria, 1837–1901, the public health movement had been living in the shadow of a dominant ‘common sense’ ideology of methodological individualism.^1^ Its populist Bible was Samuel Smiles' best‐seller of 1859, *Self‐Help*. Its intellectual taproots lay in the coming together of Benthamite utilitarianism with classical political economy along with an evangelical variant of Anglican Protestantism focused on individual salvation (Hilton, 1991). Its epoch‐creating act was the 1834 New Poor Law, whose principal goal was to end the scourge of ‘pauperism’. This was a key notion, an amalgam of political economy, utilitarianism, and soteriological evangelicalism, which expressed the idea that poverty was due to sinful, lazy, depraved and dependent individuals who needed to be disciplined and ‘saved’.

The 1834 Act engendered the ‘Dickensian’ world of the feared and hated workhouse, whose primary purpose was to motivate all able‐bodied adult males to attain independence for themselves and their families through work for whatever wages were on offer from employers in the free market economy. A future Archbishop of Canterbury, John Bird Sumner, sat on the Poor Law Commission of 1832–4 which recommended the system of ‘motivational’ (by deterrence) workhouses, but its principal architects were the holder of the first Chair in Political Economy at the University of Oxford, Nassau William Senior, and Jeremy Bentham's devoted amanuensis, the barrister Edwin Chadwick. Chadwick was also the principal architect, 14 years later, of the world's first national Public Health Act of 1848. In the intervening years Chadwick had discovered that disease was causing rising costs to his new Poor Law when family wage‐earners were incapacitated, imperilling its capacity to extirpate pauperism (Hamlin, 1998). His 1848 legislation would enable elected local authorities to tax their property owners to institute ‘sanitary’ engineering measures to radically reduce the incidence of disease through provision of abundant clean water and sewerage. This would supposedly eliminate the nuisance of disease.

Consequently, the British public health movement developed over the next half century within a statutory framework determined by the economically liberal and individualist common sense of the age. The central state was to tax as little as possible and was not to interfere in the economy and its labour markets. Keeping the urban populace healthy was a practical matter for elected local government to determine. Public health medicine was also in a formal relationship of subordination to the Poor Law in the period 1871–1919. The administration of public health statutory matters was located within a Whitehall department, the Local Government Board, whose culture was dominated by a secretariat of Poor Law officials, whose founding raison d’etre was to fight the evil phantom of pauperism (Bellamy, 1988). The Treasury aimed for tight spending controls and was intent on discouraging expensive public health measures (MacLeod, 1967, 1968).

An opportunity for re‐thinking at the highest levels of national government public policy presented itself after the military setbacks of the 1899–1902 Boer Wars. Edwardian Britain was now permeated by festering anxieties surrounding the nexus of disease, poverty, and imperial prowess (Searle, 1971). Growing calls for measures to reverse the nation's perceived decline led to a sequence of major public enquiries concerning the population's health and fitness. Central among such enquiries were those of the Royal Commission on Physical Training in Scotland (1902–03), the Interdepartmental Committee on Physical Deterioration (1903–04), and the Royal Commission on the Poor Laws and the Relief of Distress (1905–09) (cf. McBriar, 1987; Szreter, 1996).

At the epicentre of this public affair was the issue of persistently high infant mortality rates—an issue that turned into a dominant locus of ideological tension and political conflict in the new century. In 1911, a newly designed official social classification system confirmed what had essentially been known for a long time, namely that the issue was one of social inequality: in working‐class families, 133 infant deaths occurred per 1000 live births, and 152 deaths occurred per 1000 live births among unskilled labourers, compared to 77 per 1000 in middle‐ and upper‐class families (Newsholme, 1913). At stake, however, was the question of what constituted a correct scientific explanation of and, as a corollary, an adequate policy response to such striking health gaps. The two competing explanatory frameworks that shaped the public debate were, on the one hand, the hereditarian, eugenicist view, spearheaded by Karl Pearson, according to which high infant mortality weeded out the weakest members of society and led to a healthier population in the long run, thus rendering any immediate public health intervention redundant (Szreter, 1996: ch. 4; Eyler, 1997, pp. 299–305); and on the other hand, the environmentalist view—espoused, among others, by an increasingly cohesive body of public health professionals nationwide—according to which high infant mortality was socially caused and hence preventable through collective social action.

It was in this context that Arthur Newsholme, Medical Officer of the Local Government Board—a position broadly comparable to the Chief Medical Officers in contemporary England, Scotland, Wales, and Northern Ireland—came to develop what was distinctly unusual for his time (and, one might argue, still today), namely a sociologically refined, multicausal understanding of health inequality that set an important precedent for the still embryonic realms of public health and social medicine. Between 1910 and 1916, Newsholme published five book‐length official reports on infant, child, and maternal mortality which should be understood against this distinctive historical backdrop, wherein politically invested lines were increasingly drawn between socially differentiated ‘fitness’ and questions of ‘national efficiency’. Of these five reports, the third, published on the cusp of the First World War in 1914, was centred on the industrial towns of Lancashire where infant mortality rates were often significantly higher than the national average (Woods et al., 1988a, 1988b; Eyler, 1997, p. 388). Newsholme's reports differed from those produced by the Statistical Department of the General Register Office (GRO) (see below) both in scale and in its use of first‐hand observations by local Medical Officers of Health to supplement the analysis of vital registration data. In the case of Lancashire, Newsholme even went a step further by sending three of his Medical Inspectors to investigate environmental conditions in seven select towns, namely Burnley, Colne, Farnworth, Nelson, Stretford, Widnes, and Wigan. In addition, Janet Lane‐Claypon, Assistant Medical Inspector, was sent to assess preventive measures, interview local Medical Officers of Health, and accompany health visitors to the homes of infants in all the Lancashire boroughs and urban districts outside those seven visited by her colleagues (Eyler, 1997, p. 298).

## 3 UNDERSTANDING INFANT MORTALITY: FROM BRIGHTON TO LANCASHIRE

In approaching the issue of ‘national efficiency’ and the challenge posed by the hereditarian view on health inequality, Newsholme drew extensively on his prior experience as the first full‐time Medical Officer of Health of Brighton, a position he had held between 1888 and 1908. The post of Medical Officer of Health, statutorily responsible for the health of an entire population, had first been funded by the city of Liverpool from 1846, shocked at its dire mortality statistics, graphically portrayed by William Farr from newly available data collected by the GRO (Szreter, 2005, pp. 10–11, 119–19, 244). London vestries and a few other cities followed suit but it was not until the 1875 Public Health Act that parliament determined that the whole country should be divided into sanitary authorities, each with an appointed Medical Officer of Health. Dedicated diplomas for aspiring Medical Officers of Health in sanitary science, epidemiology, and bacteriology began to be offered (the first by the universities of Dublin and Cambridge) and a nationwide professional Society of Medical Officers of Health was in existence by the 1880s with its regular journal, *Public Health*, publicising new local initiatives and their results and campaigning for these posts to be made full‐time and properly remunerated (an important condition of service not addressed by the 1875 Act) (Hardy, 1993; Porter, 1991).

Within months of taking over the new full‐time post in Brighton, Newsholme had produced a report on infant diarrhoeal deaths (Newsholme, 1888), prefiguring the five more extensive Lancashire studies that would appear between 1910 and 1916. This early work emphasised the concentration of infant mortality in the unskilled segment of the working class but fell short of explaining the socially patterned distribution of ‘uncleanliness, personal and domestic, as exist chiefly among the poorer classes’. His report highlighted the role of ‘the contents of imperfectly or infrequently emptied dust‐bins, accumulations of foul manure […], imperfect scavenging of side streets, as well as putrefactive changes in food and milk (and in the feeding bottles used for infants)’ in aiding ‘the conditions under which Diarrhoea thrives’ (Newsholme, 1888, p. 3).^2^

To address the problem of socially concentrated infant mortality, Newsholme launched a local campaign to teach the working classes ‘proper behaviour’ in the form of a pamphlet, printed in tens of thousands of copies, prepared for household distribution on the prevention of disease, detailing sanitary measures (drainage, ventilation, general cleanliness, waste disposal) and stressing the importance of infant care (bathing, avoiding exposure to infected persons, and avoidance of contaminated milk, ideally through breastfeeding). The issuing of this pamphlet was complemented by regular household inspections of drainage and ventilation (which were the responsibilities of landlords) coupled with observations of, and interventions in, matters of housekeeping and domestic behaviour. To a great extent, then, this early approach placed responsibility for mortality prevention on families and households and was geared towards parental education efforts. It formed, in Newsholme's mind, the ultimate step ‘to complete in the private sphere the sanitary revolution begun in the public sphere’ (Eyler, 1997, p. 53; also pp. 299–305, and Szreter, 1996: ch. 4).

This behavioural intervention approach, and especially the household inspections, did at times incite resentment in working‐class neighbourhoods. Although Newsholme defended it as a necessary step towards social amelioration, wedding the hygienic with the ‘proper’ or ‘decent’ (*ibid*: 56), he came to acknowledge that, to be fully effective, the approach had to be complemented by special municipal services, in particular municipal waste collection (*ibid*: 59–60). Weekly garbage collection services began in Brighton in the spring of 1892, upped to twice a week from 1899, and were funded by loans sanctioned by the Local Government Board to construct a municipal incinerator. The key to preventing infant deaths, Newsholme believed, was better sanitation in and around homes coupled with changes to infant feeding practices (*ibid*: 58).

Based on the observation that disease incidence was highest at about 6 months of age, around the time infants were commonly weaned from breast to bottle, and that bottle‐fed babies were far more likely to get ill than breastfed babies, Newsholme (1902‐3) was one of several Medical Officers of Health becoming increasingly convinced at this time that infant feeding was at the root of epidemic diarrhoea and preventable infant mortality more generally (Fildes, 1998). This has been vindicated by later research (Atkins, 2019). Worried about the contamination of milk, whether on the farm, during delivery, or inside the home, he advocated, as early as 1895, the establishment of a milk depot in Brighton where mothers could purchase sterilised milk. He also changed the ordering of messages in the handbill pamphlet circulated to households, now placing infant feeding at the centre, followed by a shorter message on environmental sanitation. Additionally, he prepared a special pamphlet on infant feeding and, from 1903, began to gather systematic information on infant feeding practices across all households (Eyler, 1997, p. 62).

It was in this jointly scientific and administrative labour that Newsholme began developing a multicausal understanding of the issue at hand, recognising that the contamination of milk was a problem that intertwined behavioural and environmental causal components. It took years, even decades, until he underwent an intellectual ‘conversion’ (as he himself subsequently saw it) around 1903–04, away from a resolutely individualist moralism—an amalgam of conventional liberal Victorian ideology amplified by his own Wesleyan Methodist upbringing—in favour of a broader collectivist view of the need for public health reform (Szreter, 1996, pp. 232–4, 254–5). By then, he understood the special risks faced by the poor, who lived in crowded spaces, close to waste and faecal dust, lacking adequate facilities for storing and preparing infant food. Many of these circumstances were evidently beyond the control of any individual and hence, Newsholme argued, a larger share of responsibility had to be carried by local authorities. He thus discarded the notion that infant feeding was a purely private matter or that parental education was a complete answer—one that subtended another popular approach at the time, namely blaming working‐class mothers and their putative fecklessness with respect to child rearing (Dyhouse, 1978). As the older, more radicalised Newsholme would later observe, this had become ‘a comfortable doctrine for the well‐to‐do person to adopt; and it goes far to relieve his conscience in the contemplation of excessive suffering and mortality among the poor’ (Newsholme, 1916, p. 64).

In developing this line of reasoning, Newsholme was contributing to a broader intellectual current in the new century that was gradually upending the predominant Victorian causal understanding of the relationship between disease and poverty, namely that the former causes the latter. Outside government, the economist John Hobson famously probed the institutional mechanisms, embedded in the capitalist organisation of market exchange, that generated poverty (Hobson, 1909, 1913). Leading social investigators, such as Seebohm Rowntree, Sidney and Beatrice Webb, the young R.H. Tawney, and William Beveridge were all producing important empirical studies demonstrating the poverty‐inducing consequences of the unregulated economy and the inadequacy of the 1834 Poor Law to prevent indigence leading to family misery and even tragedy.^3^ The 1908 Old Age Pensions Act, the minimum wages imposed by the 1909 Trade Boards Act, the 1909 Labour Exchanges Act, and the 1911 National Insurance Act were all to be tangible results, enacted by a New Liberal government now prepared to intervene in the economy and the labour market, partly in order to fend off electoral pressure from the rise of the new Labour Party.^4^

By considering the possibility that poverty could also cause disease, the public health movement not only subverted the hereditarian emphasis on the ‘inherent defects’ of the poor, impervious to intervention, but also related the issue of health inequality to distal modes of social and economic organisation. Newsholme's was of course only one voice among many that served to effect this conceptual shift. We are focusing here on him because of the leading role in central government that he came to hold and because of his own candour about his changing views. Probably the most impressively rigorous study of infant mortality was in fact carried out in 1908 by Dr. John Robertson, Medical Officer of Health for Birmingham 1903–27.^5^ Already as Medical Officer of Health for Sheffield between 1897 and 1903, he had, with impressive resolve and acumen, driven through to parliament an innovative bill to make tuberculosis compulsorily notifiable in Sheffield (and so subject to free, preventive treatment), managing to skilfully side‐step the reflex resistance of the Local Government Board officialdom to any measure which they suspected of giving able‐bodied men a pretext to avoid work (Mooney, 2015, pp. 159–62). Another public health leader, James Niven, Manchester's Medical Officer of Health, explicitly linked the interaction between casual labour and trade cycles with poverty and disease in industrial cities (Niven, 1910).

This sociologically informed understanding of health and inequality did in many ways mark a return to, or a revamping of, ‘the social theory of disease’ associated with William P. Alison, the eminent Scottish doctor and social reformer, the notion of *hygiène publique*, spearheaded by the French economist and physician Louis‐René Villermé, and the social medicine of the Prussian doctor and politician Rudolf Virchow.^6^ These ideas began penetrating the British public sphere already at the very dawn of the public health movement's rise in the 1840s but had failed to shake the hold of classical political economy and were excluded from public health policy by Edwin Chadwick's project, which successfully diverted the public health movement's attention away from questions of social inequality (Hamlin, 1998). Instead, Chadwick launched his ‘Sanitary Idea’—a politically anodyne engineering solution to the crippling effects of ‘miasmatic’ disease mortality—through his *Report on the Sanitary Condition of the Labouring Population of Great Britain* in 1842, leading to the Public Health Act of 1848.^7^ The complex confluence, over several decades, of multiple institutional processes, which we detail below, was needed before the public health movement could return to and discursively articulate a sociologically grounded view of health and disease.

However, this renewed understanding after 1900 of the poverty‐disease nexus was not a mere reversal of causal arrows between the two terms of the equation. What rendered Newsholme's analysis of infant mortality more sociologically refined was its attention to fine‐grained differences across social and physical space. That mortality was tied to the life of the urban poor was evident, but empirical observations made it clear that the issue was not simply reducible to matters of income or wealth. He noted early on that miners, who were among the best paid workers, had unusually high household infant mortality rates (see Eyler, 1997, pp. 306–7). The industrial towns of Lancashire, with their many similarities and several notable differences, both with respect to ‘exposure’ and ‘outcome’, provided a kind of natural laboratory to probe the social causality at work in the making of differentials in infant mortality. Once Newsholme had taken over as Medical Officer of the Local Government Board in 1909, he was in a unique position to put this emergent model of social causality to the test.

Lancashire had been one of the main early loci for the transition to an industrial economy in Britain. The importance of the cotton industry in this economic history is well‐documented (e.g., Ashton, 1948, pp. 31–4, 70–75, 112–18; Griffin, 2018, pp. 54, 65–69, 78–81; Landes, 1969, pp. 41–2, 82–3). By 1914, Lancashire's economy was a complex interconnection of industries. Other than cotton, these included coal, chemicals, glass, iron, steel, manufacturing, and engineering (Cole & Postgate, 1949, pp. 36–38; Newsholme, 1914, p. 16). The towns of Manchester, Liverpool, Preston, St. Helens, Wigan, Bolton, Blackburn, and others were part of a complex division of labour. The industrial structure reflected not only particular local natural resources like coal and salt, but also the trade with North America and the Indian sub‐continent through the ports of Liverpool and Manchester (from 1894, when the Manchester Ship Canal opened). The Local Government Board report of 1914 comments extensively on the industrial shape of the county, the towns which were the foci of the investigation, and the impact of that industrial structure on the population:

The advent of the cotton industry caused the […] abundance of remunerative employment for both men and women, and a special concentration of the population under urban conditions which became essential in order to bring the people in close proximity to the places where they worked. Unfortunately, this has been accompanied by grave drawbacks, which include inferior housing, over‐crowding, and other defective sanitary conditions

(Newsholme, 1914, p. 6).

The report notes that by 1914, male employment was dominated across the county by textiles and mining, while textiles formed the principal source of employment for women. Wage rates were relatively high for women according to the report, with average female earnings of 25 shillings a week, though men earned more. Despite relatively high rates of employment and wages, and the regular nature of employment in the mills, what puzzled Newsholme was the fact that infant mortality was relatively high but very variable between the seven towns, with Nelson, Colne, and Stretford below the national average, but the other four towns exceeding the national average by a considerable margin (see Table 1). He was convinced that the key to understanding inequalities in infant mortality lay in the careful dissection of such geographical variation.

**TABLE 1:** Infant mortality per 1000 live births in 1913 (Newsholme, 1914, p. 9).

Geography	Infant mortality rate per 1000 live births
England and Wales	109
Burnley	174
Colne	101
Farnworth	215
Nelson	91
Stretford	91
Widnes	132
Wigan	179

The principal causes of infant deaths were identified in the report as prematurity and congenital defects, injury at birth, lack of breast milk, atrophy, debility, marasmus (extreme malnutrition), diarrhoea, enteritis, bronchitis, and pneumonia (*ibid*: 65). These in turn were linked by Newsholme to patterns of working life, domestic arrangements, routines of childcare and breastfeeding, sanitary conditions, and housing. He focused particular attention on the health of the female mill workers and especially the impacts of everyday lives on the health of the women and their babies. Specifically, he drew a connection between the patterns of employment and the knock‐on effects to domestic life and health. In effect, the report highlights the causal effects of the labour process under industrial capitalism on infant mortality rates, as mediated by two central parameters of everyday life: childcare (including breastfeeding) and material living conditions (including housing, sanitation, and food storage).

The report explicitly relates the manual work of these women to the quality and nature of childcare. In particular, the taxing nature of the work is viewed as a determinant of the relation between mother and infant and, consequently, as a determinant of mortality risk:

It is reasonable to believe that the industrial occupation of women, in so far as it exposes the pregnant mother to laborious work and strain, and in so far as it separates the infant from its mother, thus not only preventing suckling, but also diminishing the individual care which the mother can devote to her infant, must tend to increase infantile sickness and mortality. […] [The] industrial occupation of women, whether married or unmarried, may be regarded as to some extent inimical to home‐making and childcare […]. It is noteworthy that in Burnley, Colne, Nelson, and Farnworth more than seven‐tenths of unmarried females are industrially employed, and in Stretford and Wigan the proportion thus occupied is also high […]. [On] the feeding of 2,146 children at the end of six months, […] 36.4 per cent were breastfed, 33.8 per cent had mixed breast and artificial feeding, 27.4 per cent were artificially fed, and as to 2.4 per cent, there was no statement. […] Data collected by various health visitors, and shown to me on my visit, as to the method of feeding of the infants who succumbed to diarrhoea, show the already well‐known fact that it is the bottle‐fed infants which die in a proportion out of all comparison to those fed upon the breast

(*ibid*: 19–20; 138–140).

‘Free labour’ under industrial capitalism, whereby workers, ‘under the compulsion of the whip of hunger’ (Weber, 1924, p. 277), sell their labour power, thus emerges as a major structuring force of everyday life, along with the broader material determinants of infant mortality which are also vividly depicted. Ranging from the insanitary nature of toilet facilities to the preparation and storage of food, the material substrate of everyday life emerges as a powerful vector of disability, disease, and death:

The excreta pails are emptied into large brick tanks and covered with sifted ashes…The contents of these tanks […] are extremely offensive […]. Not only is the deposit of such *large quantities of excremental refuse* attended by most offensive stenches, but *flies are bred here* in such quantities that, as I am informed, in the summer time the walls and chimney […] are black with them. […] Proper food cupboards exist in but few of the working‐class houses in Widnes, and where there are cupboards they are often unventilated. In most houses, food, milk, &c., have to be placed on shelves in the kitchen or scullery, and are thus *exposed to contamination of various kinds*. […] Usually there is no place for the storage of food, including milk, other than a small cupboard placed at the side of the kitchen fireplace, and possessing no means of ventilation to the outside air […]. [There is a] general absence of proper food cupboards in the working‐class houses of Wigan, Stretford, Widnes, and Farnworth

(pp. 80–81, 14, 114; emphasis added).

A notable feature of this lifeworld is where the material conditions meet the distinctly gendered nature of domestic labour, whereby women, despite their reliance on long hours of wage labour for subsistence, are nonetheless exclusively responsible for childcare, sanitation, and food preparation:

The conditions arising in many households consequent upon the return to work of the mothers leave much to be desired. By working early and late many of the women maintain a fair standard of cleanliness and order in their homes. *The strain on a woman who is at work in the mills all day and has to do the household cleaning and washing either before she goes out or when she returns, from work must be very considerable, and can hardly be conducive to health*. As a matter of fact the cleansing of the house must frequently be left for Saturday afternoon or Sunday morning. […] The mother who is at work all day will very naturally cook as little as possible, and the family food must be purchased at one of the numerous shops where commodities can be obtained ready prepared. These will include potted or tinned foods, of other preparations calculated to excite a desire for food in those returning home after long hours spent in air which is far from fresh. The food which is purchased ready prepared is, moreover, more costly than that which is cooked at home, and calls for a larger family income

(pp. 138–9; emphasis added).

In light of these findings, Newsholme firmly believed and effectively demonstrated that infant mortality was ‘both a sanitary and a social problem involving both individual and community responsibility’ (Eyler, 1997, p. 307). Retreating from this responsibility was nothing less than ‘an unsocial negation of civilisation’ (Newsholme, 1908, p. 189). While acknowledging the important role of mothers and of domestic practices more generally, he eschewed the facile path of identifying maternal ‘ignorance and fecklessness’ as the root cause of the problem (see Eyler, 1997, pp. 310–16). He saw no reason for viewing working‐class mothers as more ignorant or feckless than mothers in other social classes, but pointed out that in situations of serious deprivation, ignorance and mistakes are more tragically consequential. As shown in the report, working‐class mothers were more likely to work outside the home and hence be physically and mentally exhausted, all while their homes were also more likely to be overcrowded and insanitary.

Against this backdrop, Newsholme described the high infant death rate in the industrial towns as the result of ‘inexcusable municipal neglect and parsimony’ (Newsholme, 1910, p. 344). His wide‐ranging investigations had undermined the hereditarian dismissal of public intervention by providing a cogent alternative account, backed by detailed empirical data. However, he also devoted his first infant mortality reports to attacking the eugenicist argument more directly. Mobilising statistical outputs from the GRO (see below), he demonstrated that high infant death rates—which, according to the hereditarian view, would weed out the population's ‘weak stock’—did not produce a healthier population in the form of lower rates of mortality in succeeding years at higher ages:

If natural selection during infancy […] has left a juvenile population less prone to disease, the effect of this selection is most effectively and completely concealed by the evil environment […] which causes the hypothetically stronger survivors to suffer from excessive mortality. […] There need therefore be no hesitation in making every practicable effort to reduce infant mortality

(Newsholme, 1910, pp. 17–18).

This line of argument led the hereditarian model to be viewed by the medical community as having ‘received a severe check, if not been actually destroyed, by the pains‐taking and scientific investigation of Dr. Newsholme and his department’ (Ashby, 1915, p. 11). But even before Newsholme's reports saw the light of day, environmentalists had already succeeded in shaping the Physical Deterioration Committee's summary conclusion that the influence of heredity was ‘not a considerable factor in the production of degenerates’, despite the Committee's origins having been the product of public concerns more in line with hereditarian views.^8^ This conclusion was deeply informed by a full‐fledged social class analysis furnished by the Board of Education's Chief Medical Inspector of Special Schools, Alfred Eicholz. Eicholz’ analysis connected theory and data to forge a novel understanding of social stratification and the causal mechanisms of inequality (Szreter, 1996, pp. 211–15).^9^ Also of considerable importance was the testimonial made by Miss A.M. Anderson, HM principal Lady Inspector of Factories, who, much like Newsholme in his coming reports, attested to trans‐ or supra‐individual causal forces connecting bad feeding, overcrowding, factory employment of mothers, and infant mortality (*ibid*: 217). The Committee's report precipitated a growing movement towards greater acceptance of collectivist principles and new emphasis on economic and cultural forces that trapped individuals in situations of poverty (cf. Searle, 1971; Szreter, 1996, chs.4–5).

Newsholme's empirical corroboration of and continued emphasis on the environmentalist understanding of infant mortality served not only to puncture the rise of social Darwinism but also weakened the other dominant view at the time, namely that high infant mortality was simply caused by female employment and hopelessly uneducated working‐class mothers (Dyhouse, 1978; see also Dwork, 1987). The penetration of this latter view into scientific discourse was (at least in part) related to George Newman's influential 1906 book, *Infant Mortality: A Social Problem*, which in many ways set an alternative agenda for research on infant mortality in the twentieth century, which Newman subsequently favoured upon becoming the nation's Chief Medical Officer in the newly created Ministry of Health in 1919. It marked a shift in emphasis away from ‘environmental conditions’, a staple of the public health reformist movement of the preceding decades, towards the ‘social’—a term which was laden with ideologically ambiguous meaning. More specifically, Newman's understanding of the ‘social’ was anchored in an individualist obsession with maternal micro‐behaviour and responsibility rather than any veritable concern with the ambient social space in which such behaviour took place. Whereas Newsholme's model, after his 1903–04 ‘conversion’, eschewed such moralising individualism in favour of a more collectivist view, Newman was something of a Victorian throw‐back, who seemed not to have absorbed the evidence of Newsholme's Local Government Board reports.^10^ However, it should be noted that Newman's continuing interwar preoccupation with motherhood's responsibilities was related to a pervasive, gendered blind spot in British social policy, dating back to 1834 (Thane, 1978).^11^

## 4 HISTORICISING NEWSHOLME'S MODEL: FOUR INSTITUTIONAL PROCESSES

To appreciate fully the novelty of Newsholme's multilevel model of infant mortality, one must view it in its proper context: as the culmination of a long historical sequence—dating back almost a full century—involving a continual interplay between scientific development, ideological change, and political conflict in the era of Victorian liberalism. This sequence was mediated by four key institutional processes that we construe as organised counter‐movements to the joint effects of rapid economic growth and the New Poor Laws instituted in 1834 (Szreter, 1997). In the Victorian era, major societal convulsions unfolded in the wake of rapid industrial transformation, causing the breakdown of nationally shared systems of meaning and collective representation across social classes, of recognition and reciprocity, of security and stability. This was aggravated by the 1834 New Poor Laws, which supplanted the comprehensive safety net previously offered by the Elizabethan Poor Laws (Cooper & Szreter, 2021: ch. 11; Slack, 1995; Szreter et al., 2019). As mentioned above, the New Poor Laws instead introduced a deterrent mode of poverty management epitomised by the infamous Victorian workhouse.

The first organised reaction to such ‘social sundering’ (to borrow Therborn's [2013, pp. 21–28] phrase) was institutionally embodied by the GRO, the organisation set up by statute in 1836 to administer the new civil and vital registration system of England and Wales and put in charge of the decennial censuses from 1841 onwards. William Farr, its first statistical superintendent, pioneered life table comparisons as a gold standard for the Victorian public health movement and employed them to publicise the nation's urban health problems (Eyler, 1979). As new social convulsions unfurled across the nation, we now know that aggregate population health gains that had accumulated since the 1730s ground to a halt in the 1820s, whereafter the national life expectancy trend remained stagnant for about half a century until the 1870s (Wrigley & Schofield, 1981; Wrigley et al., 1997, tab. A9.1, p.614). Beneath this aggregate trend, and despite rising disposable income, was a serious deterioration in health during the second quarter of the nineteenth century for those segments of the population directly involved in urban and industrial expansion, including a dramatic rise in infant and especially in child mortality in industrial townships across the country (Kitson, 2014; Szreter & Mooney, 1998; Woods, 1993; Wrigley et al., 1997). Rapidly growing large industrial cities had life expectancies at birth of 34–37 years in the 1850s, with life expectancy in Liverpool and Manchester as low as 31 and 32 years, respectively. These numbers only increased to 38 and 36, respectively (and 40 for most others), by the 1890s when national life expectancy at birth stood at 46 years (Szreter & Mooney, 1998: tab. 2 and 8).

Within a few years, thanks to Farr's indefatigable activity,^13^ the GRO had morphed into a major social and economic intelligence system that balanced science production and public health propagandism in its annual publications. Not only did it provide much‐needed factual information about patterns of mortality across the nation, but it also emphasised geographical differences, monitored trends over time, and made international comparisons with other major European cities. Notably, this relentless information war led to Section 8 of the 1848 Public Health Act, stipulating the need to establish local health boards to implement local sanitary reforms if annual mortality rates were found to be above the national average. Moreover, Farr went on to develop the concept of ‘Healthy Districts’ in the 1850s as the desirable standard to which local authorities should aspire and published league tables of cities ranked by their death rates—both strategic and potent rhetorical inventions that induced local competition and rivalry. By the end of the 1850s, the Parliament could no longer stand the stench of the Thames, producing the flat‐rate‐funded Metropolitan Board of Works which funded and built Bazalgette's famous sewers system (Szreter, 1991a, p. 496). But in the provinces, similar policies had to await the combination of the1869 municipal franchise changes—giving the majority of votes for the first time to non‐rate‐paying voters—plus the advent of ‘gas and water socialism’ to fund municipal improvements out of natural monopolies (see below). Farr's successors at the GRO ensured that they continually upped the levels of salubrity for which such municipal rivals were aiming, thus intensifying the competition for improving the people's health (Szreter, 1991b).

At the same time, the public health movement was operating in an era of Victorian libertarianism, whose dominant policy orientation was captured by John Stuart Mill's adage according to which *laissez‐faire* ‘should be the general practice: every departure from it, unless required by some great good, is a certain evil’ (Mill, 1848, p. 950). Negative attitudes to the central state and government were anchored in eroding social trust and precipitated administrative decline. The rising electoral dominance of a newly enfranchised petty capitalist class—socially and economically located just above waged workers, aspiring to flee the ‘inner city’ to join the elites in their suburban villas—used their ratepayers' association to mobilise against municipal public health action, which would cost them dear (Fraser, 1976; Hennock, 1963). This deep‐seated ideological resistance to any form of collectively organised interventionism that could inconvenience capital and property owners, notably in the form of local taxation, stymied the public health movement for a generation after the 1830s since it was constrained to seek devolved solutions to the nation's health crisis (Hanley, 2016).

Coupled with, and fuelled by, the GRO's data‐driven propaganda strategy, this fostered a second organised response in the form of the so‐called municipal ‘civic gospel’, a religiously inflected civic consciousness preached since the 1840s and first acquiring influential adherents in the 1860s among neo‐patrician leaders of the city of Birmingham (Szreter, 1997; see also Jenson, 2009). Its most famous proponent was Joseph Chamberlain, a major industrial magnate and mayor of Birmingham between 1873 and 1876, who was positioned at the centre of a large network of leading business families and non‐conformist congregations that brought people together in Birmingham across social and religious divides (the classic study is Hennock (1973); see also Hunt (2004): ch. 8) Politically, the movement operated through the local Liberal Party, creating an innovative ward‐based electoral machine to mobilise the newly enfranchised working class, following the 1867 Second Reform Act and the 1869 Municipal Franchise and Assessed Rates Acts. This gave rise to a unique configuration of cross‐class political alliances that forged a novel discourse of locally rooted civic pride, recasting municipal investment as investment in the populace itself. This led to a distinctive rhetorical innovation that construed true ‘economy’ and ‘efficiency’ as consisting in the creation of a healthier, more skilled, educated, productive, and competitive workforce and citizenry (Szreter & Woolcock, 2004). However, the movement's rhetoric sought to avoid unnecessary provocation of ratepayers' ire and treated property rights and fiscal sensitivities with respect. To this end, practical innovations that took account of social tensions and divisions were devised, most notably via the use of long‐term, low‐interest loans to buy up productive natural monopoly services in the city, especially for basic utilities like gas, water, and electricity, thereby massively raising revenue streams, independent of the property rates, to fund social and health services in the city (Szreter, 1997).

Following Chamberlain's personal brokering of a massive innovative loan to purchase Birmingham's gas works, the Treasury decided it would be better to manage the process of further municipal buy‐ups of local monopolies itself. Thus, it made much more viable the process of obtaining subsidised loans for municipal sanitary investments from the central Exchequer, which had been possible in theory since the 1848 Public Health Act. The scale of such loans totalled £84m during 1871–97, of which £65m went to urban authorities, compared to only £11m loaned out in 1848–70 (Wohl, 1983, pp. 113, 162–63; an upward trend is also confirmed with new data by Harris and Hinde (2019)). By running these local monopoly services at a profit, regular revenue was generated for further municipal spending (Millward & Sheard, 1995; Wilson et al., 1993). Not only did per annum investments rise from £5m in 1856–71 to c. £13m in 1874–94 and to c. £30m by the early twentieth century (Feinstein, 1972, 1976); but also by 1905 local spending accounted for around one‐half of total government recurrent expenditure (Szreter, 2005, pp. 124–25; Waller, 1983, p. 264). This revenue stream supported the massive expansion of municipally employed sanitary, housing, and food inspectors and visitors, which were crucial for enforcing higher standards in the environment, policing landlords and reducing overcrowding, and regulating the building and food supply trades. For instance, when Liverpool's long‐serving Medical Officer of Health E.W. Hope retired in 1923, he had seen the number of salaried public health staff working under his direction to improve the population health of Liverpool increase tenfold since 1883 to over 470 employees. Chamberlain's innovations in Birmingham had major spillover effects on other towns and cities across the country (Bell & Millward, 1998). In the subsequent New Liberal era the value of many of the key public health measures promoted in new legislation, such as notification of all births to enable health visitors to make timely visits (1907 Notification of Births Act), free school meals (1906 Education [Provision of Meals] Act), and school medical inspections, had already been road‐tested previously by innovative municipalities. For instance, school medical inspection, adopted as a national system by the 1907 Education (Administrative Provisions) Act, began in Bradford in 1893 under Dr James Kerr, who then brought it to the LCC on his appointment as its Medical Officer of Health in 1902 (Szreter, 2005: ch. 9). Investment in a school medical inspection system was of course a vital further intelligence system for any society serious about long‐term improvement of the population's health (Harris, 1995; Welshman, 1996).

This civic movement's emphasis on the value of public service led to a third institutional movement centred on the professionalisation and organisational embedding of ‘public health’ as a distinctive category of state policy and public discourse. It was in this same era that a new cadre of public health professionals became durably embedded in the institutional fabric of the public sphere, most notably in the form of the new post of Medical Officer of Health, which was established as a salaried statutory office in every authority after the 1872 Public Health Act. There was a simultaneous proliferation of related roles, including sanitary engineers, surveyors, food and drug analysts, sanitary inspectors, and town clerks. *Public Health*, the professional and academic journal founded in 1888, the *Journal of the Sanitary Institute* from 1894, and *The Journal of Hygiene*, established in 1901, provided fora for intellectual exchange and professional consolidation. This process yielded an increasingly well‐organised and cohesive body of public health professionals nationwide with a growing sense of solidarity forged through national associations (Szreter, 1996: ch. 4).

These organised bodies, largely composed of local authority‐employed public service professionals, would come to harbour the most prominent voices surrounding issues of urban poverty and health during the Edwardian ‘national efficiency crisis’. Professionally trained experts with a wealth of empirical and administrative experience, as we have seen in Newsholme's case, were now uniquely positioned to attest to the effects of poverty and the true causes of ‘impairment’ and ‘degeneration’ that were lamented by hereditarians. Prominent examples include John Tatham—Medical Officer of Health for Manchester and Salford (1888–93) and Farr's second successor at the GRO (1893–1909)—who gave crucial evidence to the House of Lords Committee for the Bill for the Better Protection of Infant Life in 1897, and again for the 1904 Inquiry into Physical Deterioration, as did the aforementioned Alfred Eicholz (an HMI of Schools at the time), whose wide‐ranging social‐class analysis set the parameters of future inequality research. By recasting both poverty and disease as resulting from preventable environmental deprivations, these voices successfully forged a novel collectivist language for addressing the nation's social problems and thus initiated a joint offencive onto the national political arena at a time when the Liberal Party was coming under severe pressure on its left flank from the newly formed (in 1900) party of working‐class men, the Labour Party (cf. Reid, 2004, pp. 259–66).

The fourth and final institutional process of great significance was legislative (cf. Szreter, 1988). As noted earlier, the 1832 Great Reform Act and the 1835 Municipal Reform Act had created a new electorate of small‐scale property owners and, in so doing, had driven a wedge between urban employers and wage labourers who were left on opposite sides of the electoral divide,^14^ thus enhancing antagonistic class relations. The result was the so‐called ‘shopocracy’, a commercial petty bourgeoisie that dominated the country's electorate, both at national and local authority levels, bent on low national and local tax rates and, by extension, allergic to the reformist movement's call for collectively organised and collectively funded public health intervention. In contrast, the 1869 Municipal Franchise Act and Assessed Rates Act suddenly increased the proportion of urban voting adult males from an average of 35% in 1865 to 57% in 1871. This legislative change, then, had served to quadruple the size of the urban local government electorate, now comprising almost two‐thirds of proletarian men—those who had been left out in the 1830s. Statistical analysis by Aidt et al. (2010: tab. 1) has shown that in those towns where electorates rose above 40%, this was strongly associated with increased public health expenditure from the early 1870s. Thus, urban politicians were stimulated to follow Chamberlain's lead and recalibrate their electoral strategies, to take account of a wider range of interests, now including the non‐ratepaying manual working classes.

Meanwhile, the legal basis for collective action through municipal government was set by the 1871 Local Government Board Act, which created a unified department of state for local government to be led by a cabinet‐rank minister; the 1872 Public Health Act, which established a national network of local sanitary authorities and specified statutory duties of local authorities, including, for instance, the duty to ensure a clean water supply; and the consolidating 1875 Public Health Act, which further specified the public health functions and statutory duties of local authorities. This threefold basis emerged in the wake of recommendations of the 1869–71 Royal Sanitary Commission, which had found that Chadwick's 1848 blueprint for a sanitary engineering revolution in Britain's towns and cities had failed to materialise for lack of political and economic will.

In short, the public health movement's ability to develop and articulate a sociologically informed understanding of health inequality had its roots in the continuous interplay between four key institutional processes, as embodied by the GRO, by the municipal ‘civic gospel’, by a nascent body of public health professionals, and by attendant legislative changes. These processes—though never fully unitary and clearly delineated—generated the conceptual, analytical, and organisational tools that were needed to successfully construe inequalities in infant mortality through the lens of social causality and thus to gradually dislodge Victorian individualist ideology.

## 5 CONCLUDING DISCUSSION

This paper has related the process by which Arthur Newsholme and the British public health movement, in the post‐Victorian decade before the Great War, sought to render scientifically intelligible, by way of a sociologically informed mode of reasoning in relation to carefully collected evidence, the issue of infant mortality and health inequality. Newsholme's Lancashire study offers not only a vivid portrayal of (variations in) working‐class life under industrial capitalism in Lancashire but also pinpoints the multilevel causal mechanisms, from the macroeconomic to the molecular, underpinning high rates of infant mortality. In the 1914 report, we see an interweaving of labour market working conditions, the practices associated with childbirth and breastfeeding, infant mortality, diarrhoeal infection, and sanitation.^15^ This is a mix of shared social practices which are embedded in the material circumstances and the experience of everyday life. The report also provides a graphic empirical description of the ways that young women lived and worked, the pressures on them to keep working up to and after childbirth, the consequences for them and their offspring of the nature of the mill work, and the consequences of their rapid return to work on rates of breastfeeding (cf. Beier, 2008; Pooley, 2010; Shove et al., 2012). Female employment with long working hours made breastfeeding difficult, both with respect to having the time to breastfeed and because of the flow of breast milk more easily drying up. In high‐mortality settings, breast milk was regularly replaced with commercial products using bacteriologically unsafe cow's and formula milk (cf. Atkins, 2019; Buchanan, 1985; Fildes, 1998; Galley, 2023; Morabia et al., 2013; Morgan, 2002; Szreter, 1988). Newsholme noted that in the poorest households, there were often no places to safely prepare or store infant food, which was thus easily spoilt or contaminated. Geographical differences in infant feeding and hence in infant mortality were directly related to the spatially patterned interaction between poverty, female work participation, and the surrounding system of industrial labour (cf. Reid et al., 2023).

This mode of thinking set at least four important precedents for social medicine and epidemiology in the 20th century. First, the emphasis on both individual and collective factors, on behaviours and the social context in which they take place, on choices and the myriad ways in which these could be constrained by distal forces of a political and economic nature prefigured subsequent sociological debates surrounding the interplay between ‘structure’ and ‘agency’ (Giddens, 1984). In particular, the Lancashire reports demonstrated, in their own way, that the recursive social practices of everyday life—and the ambient social space in which they unfold—are the underlying reasons why health inequalities are shaped and patterned the way they are, being (re)produced by the mutual imbrication of collectives and individuals, institutions and agents, objective positions and subjective dispositions (Blue et al., 2021; Kriznik et al., 2018). This view echoes, in embryonic form, later sociological discourses that construe societies as historically situated constellations of social positions, relations, and actions that are animated by the distribution of material and symbolic goods (Bourdieu, 1989)—distributions which, in turn, are governed by durably institutionalised networks of social power (Mann, 2012).

Second, the joint consideration of multiple possible causal factors, encompassing both material and non‐material components, presaged contemporary debates about the possible determinants of health inequality. These debates typically centre on the interplay between material, behavioural, psychosocial, institutional, and other interrelated forces (see e.g., Bartley, 2016; Berkman et al., 2014). Furthermore, in alignment with growing social and class consciousness in the era of industrial capitalism, the Lancashire reports also fed into the unification of health research and political economy—an unfinished unification that is still unfolding today (e.g., Beckfield, 2018; Birn et al., 2017).

Third, several decades later in the 1970s and 1980s, a group of epidemiologists led by David Barker began to systematically probe the question of how social disadvantage pertaining to ill health is embodied and transmitted from one generation to the next by examining historical epidemiological records, including those of the Lancashire mill towns (Barker, 1991; Barker et al., 1991; Barker & Martyn, 1992; Barker & Osmond, 1987). The goal was not so much to document the poor health of babies in the Lancashire towns as exploring the health trajectories of those who survived, as they grew into adulthood. Using the contemporary data from the Lancashire towns in the mid‐twentieth century when those babies had grown up, it was possible to trace their health by the time they reached middle age. Barker and his colleagues noted that the babies in the poorest health who survived into adulthood were also the adults with the poorest health. They argued that the explanation for this was to be found in the health of these individuals when they were in utero. This was a controversial suggestion, if viewed as mutually exclusive of proper consideration of all other influences later in the life cycle because that would seem to undermine the arguments that health inequalities are also the consequence of current exposure to wider social and economic determinants or to individual behaviours anchored in the constraints of low incomes and deprived neighbourhoods. The hypothesis developed by Barker and others is today known as ‘developmental programming’ and is certainly not viewed as exclusive of, but rather as adding to, our understandings of the pervasive harms of the poverty that is due to social inequality. A significant number of empirical analyses have now accumulated in support of the argument that what happens in utero is a fundamental influence on adult health (Burton et al., 2016), thus adding additional weight to the arguments made by Newsholme and his peers as to the importance of collectively organised preventive intervention. It is now understood that the manifold health harms due to inequality, if not redressed, accumulate at all stages of the life cycle from moment of conception onwards.

Fourth, Newsholme's approach heralded a novel mode of scientific investigation that combined careful attention to empirical detail with a sense of public purpose within a discursive framework located at the interface of social science, medicine, and public policy. As is well known, this mode of investigation has become a major tradition in the fields of public health and health policy research, as epitomised in recent decades by the work of Richard Wilkinson and Kate Pickett and by Michael Marmot's hugely influential corpus of work on the social determinants of health (Marmot, 2015; Marmot et al., 2008; Wilkinson, 2002; Wilkinson & Pickett, 2010). In sociology, the most influential paradigm that has explicitly construed health inequality through the lens of social causality is that of ‘fundamental cause theory’ (Link & Phelan, 1995).

In short, the public health movement's ability to develop and articulate a sociologically informed understanding of health inequality was derived from what its members viewed as ‘the mischief and the hindrance to real progress which are caused by adopting an empirical treatment of symptoms instead of a scientific treatment of disease’ (Newsholme, 1904, p. 1334). In probing the underlying disease rather than just the symptoms, Newsholme and others effectively granted the sociologist's greatest wish, namely ‘that the principle of causality should be applicable to social phenomena’ (Durkheim, 1982, p. 159). Thus, through an extended historical sequence involving four key institutional processes, as embodied by the GRO, by the municipal ‘civic gospel’, by a nascent body of public health professionals, and by attendant legislative changes, the public health movement successfully construed inequalities in infant mortality as a socially caused and collectively preventable failure—i.e., a social disease whose ultimate remedy was a social medicine.

Since the austerity programme was launched by the British central government in 2010, the Treasury has been tightly constraining the expenditure of local government and associated preventive health and social care activities, just as it did in the decades before 1905. Meanwhile, the percentage of children living in officially defined relative poverty has risen from 27% to 32%. These are political choices, whose harmful health consequences—and the measures necessary to prevent them—we now fully understand scientifically, thanks to the further development of the research approach which was initiated by Newsholme and his colleagues well over a century ago.

## Data Availability

Data sharing is not applicable to this article as no new data were created or analysed in this study.

## ENDNOTES

^1^
Arguably, Antonio Gramsci was the first, post‐Marxist sociologist to identify and analyse the political importance of the canons of ‘common sense’ in a society (Femia, 1987). On the characteristics of methodological individualism, see Lukes (1973).

^2^
These negative effects have since been confirmed by later scholarship: see Buchanan (1985), Fildes (1998), Morgan (2002), Morabia et al. (2013), and Galley (2023).

^3^
A Royal Commission on the Poor Laws themselves sat from 1905 but its commissioners could not agree and so Majority and Minority Reports were issued in 1909, with the result that the government did not accept either; and although the New Liberal pensions and insurance for working‐men took many out of the remit of the Poor Law, it continued as an unreformed residual service for the uninsured until the launch of the NHS in 1948 (see McBriar, 1987).

^4^
The classic study emphasising also the influence of social idealist thought is Freeden (1978). On the rising influence of the Labour Party, see Reid (2004, pp. 259–66).

^5^
This study showed that absolute income of the household was the strongest positive correlate of weight‐gain of babies over the first year of life, while the second strongest correlate was a negative one with mother resuming work soon after confinement, presumed to be the reason for early cessation of breastfeeding. However, the rise in household income brought in by a mother going back to work was actually associated with a decrease in infant mortality among this poorest section of society. See Szreter (1996, pp. 244–6).

^6^
To this list, one could also add Friedrich Engels and his landmark study of the living conditions of the British working class  (1845).

^7^
Chadwick shared with most medical authorities the idea that miasmas (noxious gases from decomposing organic matter) were the principal preventable direct source of disease; contagionism was also considered a possibility and eventually became dominant with the rise of germ theory after the 1860s (Pelling, 1978).

^8^
See Bernard Harris's careful account, which identifies how it was that this mere Interdepartmental Enquiry's lowly status—not having been endowed with the authority and resources of a Royal Commission—paradoxically allowed it to slip under the radar of the watchful conservative eyes in the Home Office—the senior Department of State monitoring the remit and terms of reference of such government enquiries—to such an extent that it took so much compelling evidence from environmentalist medical and educational professionals which informed its thoroughly environmentalist recommendations (Harris, 1995: ch. 2, especially pp. 17–24).

^9^
A second major reason for the Physical Deterioration Committee's conclusion was the exposition given to them by D.J. Cunningham, Professor of Anatomy at the University of Edinburgh, of the then‐new rediscovery of Mendelism, the implications of which were to fundamentally discredit the conclusions drawn by Karl Pearson and his followers from their ‘biometrics’ methodology. See Szreter (1996, pp. 223–5). For more on Eicholz's influence, see Harris (1995: ch. 2).

^10^
On Newman's lacklustre leadership during the challenging interwar years, in marked contrast to Newsholme's investigative approach, see Sheard and Donaldson (2006, pp. 27–8) and Webster (1982).

^11^
Charles Booth had long ago demonstrated that in East London in the 1880s, one‐third of all families depended for survival on the earnings of husband and wife, while in York in 1901 Rowntree discovered that the wages of wives and children ‘frequently amount to more than the earnings of the head of the household’. Nevertheless, the unemployment provisions of the National Insurance Act of 1911 excluded married women out‐workers from complex arrangements made for the unmarried, on the grounds that married women's earnings were not essential to the family economy. Thane (1978, p. 33) concludes that ‘the specific problems of women wage earners were ignored by the Poor Law from beginning to end.’

^13^
It is relevant that the primary goal of the Marriage and Registration Acts setting up the GRO was to provide all‐inclusive legal records of family relationships for all propertied citizens including the fast‐growing number of non‐conformists (hence this was a bill passed by the great Whig‐Liberal reforming government of the 1830s). Most legislators creating the GRO had little idea of the public health promoting functions it was to take on thanks to the partnership between Farr and his line manager, George Graham, the first Registrar‐General.

^14^
Before the 1832 and 1835 Reforms of the franchise, many major manufacturing towns and cities had no elected parliamentary representative at all. This meant that both employers and their waged workers had a common grievance (e.g., the founder of the Birmingham Political Union and leading figure for the 1832 Reform Act was the banker Thomas Attwood who later, in 1839, presented to Parliament the first Chartist petition for universal manhood suffrage). However, the Act of 1832, though giving MPs for the first time to Birmingham and 41 other urban boroughs, also only gave the vote to property owners.

^15^
The Medical Officer of Health Reports are a comparably rich source of material, which we have not delved into here for lack of space. For London, see Hardy (1993). In Lancashire, the reports of the towns of Barrow, Lancaster, and Preston have been used by Beier to examine the nature of health cultures in the three towns from 1880 into the twentieth century. They, too, provide vivid descriptions of the ways the local economic and social conditions affected the health of the urban working class (Beier, 2008). The recursive and relentless onslaught of social and environmental conditions and the ways in which these shaped human social practices are very carefully described in the Reports.
